# Analysis of disease clusters and patient outcomes in people with multiple long term conditions using hypergraphs.

**DOI:** 10.23889/ijpds.v7i3.2028

**Published:** 2022-08-25

**Authors:** James Rafferty, Alexandra Lee, Ed Bennett, Jane Lyons, Farideh Jalali-Najafabadi, Thamer Ba Dhafari, Alan Watkins, Alexander Pate, Glen Martin, Ashley Akbari, Ronan Lyons, Niels Peek, Rowena Bailey

**Affiliations:** 1 Swansea University Medical School, Swansea University; 2 Swansea University, dept. of Computer Science; 3 Centre for Genetics and Genomics Versus Arthritis, Centre for Musculoskeletal Research, The University of Manchester; 4 Division of Informatics, Imaging and Data Science, School of Health Sciences, The University of Manchester

## Objectives

Having multiple long term health conditions (MLTCs), also known as multimorbidity, is becoming increasingly common as populations age. Understanding how clusters of diseases are likely to lead to other diseases and the effect of multimorbidity on healthcare resource use (HRU) will be of great importance as this trend continues.

## Approach

Graph-based approaches, also called network analysis in the literature, have been used previously to study multimorbidity. The use of hypergraphs, which are generalisations of graphs where edges can connect to any number of nodes, and their application to the problem of understanding multimorbidity will be discussed. Analysis using hypergraphs was carried out using a population-scale cohort of people in the Secure Anonymised Information Linkage (SAIL) Databank to find the diseases and disease sets which are most important based on a measure of prevalence and measures of healthcare resource utilisation in secondary care.

## Results

The most important sets of diseases based on the centrality of a hypergraph weighted by a measure of prevalence featured hypertension, and the most important was hypertension and diabetes. The most important sets of diseases based on the centrality of a hypergraph weighted by a measure of unplanned inpatient HRU were arrhythmia, heart failure and hypertension while for a measure of outpatient HRU the most important set of diseases was diabetes and hypertension.

## Conclusion

Hypergraphs are very flexible and general mathematical objects and there is still a great deal of development that can be done to make them more useful in epidemiological settings and beyond.

